# Thyroid disorders and gastrointestinal dysmotility: an old association 

**DOI:** 10.3389/fphys.2024.1389113

**Published:** 2024-05-02

**Authors:** Guang-Meng Xu, Ming-Xin Hu, Si-Yu Li, Xuan Ran, Hao Zhang, Xiang-Fu Ding

**Affiliations:** ^1^ Department of Colorectal and Anal Surgery, The Second Hospital of Jilin University, Changchun, China; ^2^ Department of Thyroid Surgery, The Second Hospital of Jilin University, Changchun, China

**Keywords:** thyroid disease, thyroid disorder, gastrointestinal (GI) motility, thyroid hormone, thyroxine

## Abstract

Gastrointestinal motility symptoms may be closely related to thyroid diseases. Sometimes, such symptoms are the only thyroid disease-related clue although the degree of the symptoms may vary. The exact mechanism of action of thyroid hormones on gastrointestinal motility is not completely understood, however, a clue lies in the fact that muscle cell receptors can be directly acted upon by thyroxines. Both hypo- and hyperthyroidism can cause impairment of gastrointestinal motility, modifying structure and function of pharynx and esophagus, and regulating esophageal peristalsis through neuro-humoral interaction. In hyperthyroid patients, alterations of postprandial and basic electric rhythms have been observed at gastro-duodenal level, often resulting in slower gastric emptying. Gastric emptying may also be delayed in hypothyroidism, but an unrelated gastric mucosa-affecting chronic modification may also cause such pattern. Hyperthyroidism commonly show malabsorption and diarrhoea, while hypothyroidism frequently show constipation. In summary, it can be stated that symptoms of gastrointestinal motility dysfunction can be related to thyroid diseases, affecting any of the gastrointestinal segment. Clinically, the typical thyroid disease manifestations may be missing, borderline, or concealed because of intercurrent sicknesses. Motility-linked gastrointestinal problems may easily conceal a misdetected, underlying dysthyroidism that should be carefully analyzed. Here, we aim to elaborate on the associations between thyroid disorders and GI dysmotility and the common clinical manifestations associated with GI dysmotility.

## Introduction

The metabolic activity of most of the body organs is regulated by thyroid hormones (Pirahanchi et al., 2024). Hence, it is quite common to find thyroid diseases in the general population. Iodine-replete communities show spontaneous hypothyroidism prevalence of 1%–2%; it is also 10 times more commonly found in women as compared to men while older women are more likely to be affected (Vanderpump and Tunbridge, 2002). In women, hyperthyroidism prevalence is between 0.5% and 2% in iodine-replete communities; and is nearly 10 times more than in men (Vanderpump and Tunbridge, 2002). Nearly 8% women and 3% men are affected by subclinical hypothyroidism, defined by normal levels of thyroid hormones and high levels of serum thyroid stimulating hormone (TSH) (Fatourechi, 2009). In absence of TSH-secretion inhibitory medication or any diseases (non-thyroidal illness, hypothalamic, or pituitary), almost 3% of the population is affected by subclinical hyperthyroidism, defined by normal levels of thyroid hormones and low levels of serum TSH (Fatourechi, 2009). Thyroid disorders can be linked with gastrointestinal (GI) diseases or GI symptomatology (Kyriacou et al., 2015). Alternatively, non-thyroidal disorders can also be associated with GI diseases, resulting in thyroid function disruption.

**FIGURE 1:**
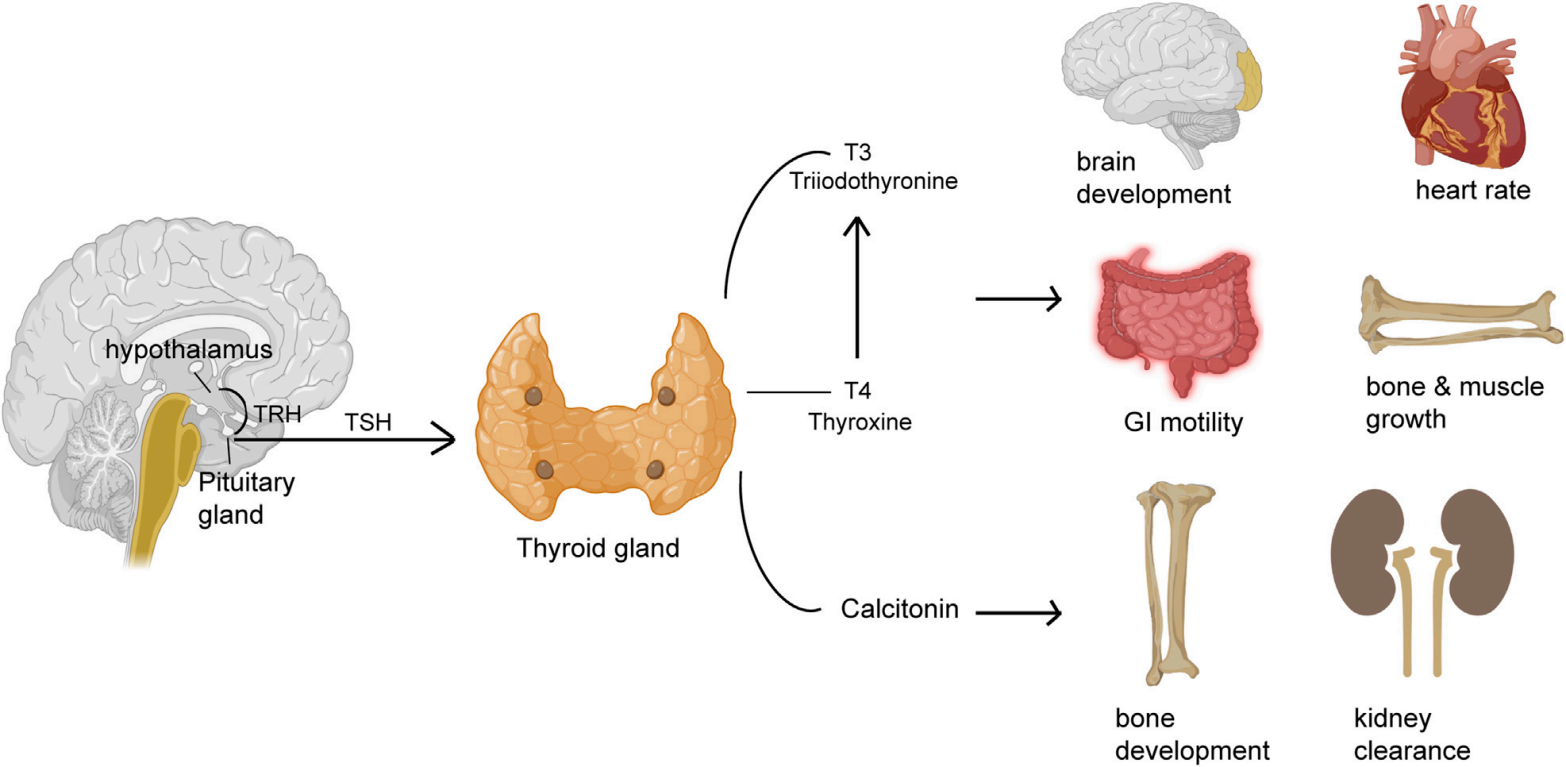
Thyroid hormones regulate the function of different organs. The pituitary gland releases thyroid stimulating hormone (TSH) that acts on the thyroid gland to release hormones like thyroxine (T4) or its active form triiodothyronine (T3) and calcitonin that regulates several different functions of different organs including GI motility, heart rate, brain development, muscle and bone growth, bone health and kidney clearance.

The small gland, thyroid produces hormones that play major roles affecting almost every organ, tissue, and cell of the body (Figure 1). The body mostly releases the inactive form of thyroid hormone thyroxine (T4). The active form of T4, triiodothyronine (T3) is released by the body in small amounts (Kyriacou et al., 2015). However, the synthesis of T3 majorly takes place in the liver, heart, brain, GI tract, and kidneys. T3 is a critical determinant of infant somatic development and adult metabolic activity (Kyriacou et al., 2015). The functions of thyroid hormones have critical control over all organs that include the GI tract via the regulation of GI motility and digestive juice secretion rates. Both hypo- and hyperthyroidism may cause deregulated GI motility and function (Table 1). Thyroid tissue destruction, inflammation, and or enhanced *de novo* hormone synthesis can lead to hyperthyroidism (Wiersinga et al., 2023). Some of the common hyperthyroidism causes include Grave’s disease, thyroiditis, toxic adenoma, administration of exogenous thyroid hormone, or toxic multi-nodular goiter (Wiersinga et al., 2023). Hypothyroidism, on the other hand is caused due to inadequate thyroid hormone production or suboptimal thyroid hormone activity at the site of action (Chiovato et al., 2019). Hypothyroidism is caused majorly because of Hashimoto’s thyroiditis, thyroidectomy, or thyroid ablation (Chiovato et al., 2019). Women and older persons are affected more than men under both the thyroid disorder conditions.

**TABLE 1:** GI manifestations of thyroid disease.

Hyperthyroidism	Hypothyroidism
Upper gut	Upper gut
• Dysphagia	• Dyspepsia
• Atrophic gastritis	• Dysphagia
• Recurrent nausea/vomiting	• Achlorhydria
• *H. pylori* recurrence	
Lower gut	Lower gut
• Lactose intolerance	• SIBO
• Diarrhoea	• IBS features
	• Colonic pseudo-obstruction
	• Megacolon
	• Constipation

The peptide hormone gastrin is mainly responsible for hydrochloric acid secretion into the stomach, increasing gastric mucosal growth, and gastric motility (Prosapio et al., 2024). Gastrin exerts a thyroid function-dependent influence on GI motility (Wiersinga and Touber, 1980). Gastrin concentrations in the serum is increased in hypothyroidism (Sagara et al., 1983). However, numerous studies show difference in concentrations of serum ghrelin levels in a thyroid function-dependent manner (Gjedde et al., 2008; Kim et al., 2015; Wittekind et al., 2021). Ghrelin is a gut-derived orexigenic peptide that controls energy expenditure and appetite (Wu and Kral, 2004). Ghrelin has been associated with enhanced rate of gastric emptying (Levin et al., 2006; Cheung and Wu, 2013). Administration of parenteral ghrelin stimulates migrating motor complex contractions thereby causing increased gastric emptying (Mekaroonkamol et al., 2022). On the other hand, reduced concentrations of active ghrelin can be associated with slower rate of gastric emptying (Levin et al., 2006). Conflicting reports have emerged regarding overt hypothyroidism-associated ghrelin concentration levels where it has been found to be either decreased (Altinova et al., 2006), increased (Gjedde et al., 2008), or not affected (Tanda et al., 2009). No study has been carried out regarding the association of ghrelin with gastric motility in response to a meal in subclinical hypothyroidism (SCH).

In this narrative review, we aim to elaborate on the associations between GI dysfunction and thyroid disorders and the common clinical manifestations associated with GI dysmotility.

## Hypothyroidism and GI motility

Almost 1.4% of the world population is affected by hypothyroidism (Chiovato et al., 2019). Hashimoto’s disease, an autoimmune disorder is thought to be the most common reason behind hypothyroidism (Lin et al., 2023). It has also been linked to other autoimmune conditions like celiac disease, primary biliary cirrhosis, diabetes mellitus, inflammatory bowel disease, and pernicious anemia (Grasberger et al., 2018; Huang et al., 2021; Rong et al., 2021; Ashok et al., 2022; Kjaergaard et al., 2022). Hypothyroidism is also caused due to thyroidectomy and earlier radioactive iodine therapy (Hu et al., 2020). The common hypothyroidism symptoms include fatigue, coarse hair, edema of eyes, face and hands, hoarseness, pallor, dry thick skin, and cold intolerance (Pirahanchi et al., 2024). The common complaints of gastrointestinal problems are nausea/vomiting, constipation, abdominal pain, and anorexia (Santonicola et al., 2019).

GI motility dysfunction develops following reduced esophageal sphincter pressure and lowered contraction amplitude in esophageal body leading to reflux and dysphagia. Thyroid disease treatment show improvement in such symptoms (Eastwood et al., 1982). In some hypothyroidism individuals, secretion of acid is diminished, a finding that can be linked to gastric mucosa changes but not with the severity or duration of the hypo-functioning thyroid (Dotevall et al., 1967). Higher cell cytoplasmic antibodies of parietal origin are associated with Hashimoto’s disease so an autoimmune gastritis may result in lower acid output (Boutzios et al., 2022). Reduced gastrin levels may also cause low acid output in some patients (Kishikawa et al., 2022). Some hypothyroidism individuals may show delayed gastric emptying (Khraisha et al., 2015). This delay is supposedly in the emptying phase rather than the solid food processing lag phase in the stomach (Kao et al., 2004). Since gastric myoelectrical abnormalities are not associated with the delay in gastric emptying, it may be a result of dysfunction in smooth muscle in coordination with the duodenum and antrum, or pylorospasm (Wang et al., 2023). Hypothyroid symptoms and dyspepsia, which are correlated, may show improvement with thyroid hormone replacement therapy. Dyspepsia is not correlated with TSH levels, radioscintigraphy or with other EGG parameters. Hypothyroidism patients may show up with phytobezoars in their stomach particularly following gastric surgery (Yang and Cho, 2021). Intestinal bezoars may cause obstruction (Bouali et al., 2021). Lactulose breath test-measured orocecal transit time are found to be normal, suggestive of the fact that slower bowel habits are not necessarily due to small bowel delay (Tobin et al., 1989). However, radioactive iodine-mediated hypothyroidism in hyperthyroid patients caused significant delay in transit time (Sheehan and Doi, 2016). Nearly 54% of hypothyroidism patients show presence of bacterial overgrowth in the small intestine (Lauritano et al., 2007). This takes place even if the enrolled patients are euthyroid in the beginning of treatment. This suggests that there is no spontaneous clearance of the bacterial overgrowth once established. Individuals with bacterial overgrowth show symptoms such as bloating, flatulence and abdominal discomfort which are improved with antibiotics. Reduced hollow viscera motility, including stomach, colon, esophagus, and small intestine, have been observed in myxedema which gets reversed with replacement therapy for thyroid hormone. This is characteristic of functional bowel disease (Shizuma, 2016). Hypothyroidism has also been linked with reduced stool frequency, ischemia, paralytic ileus, vascular malformations, megacolon, volvulus, and pseudoobstruction (Thakur et al., 2020). The distended small bowel and colonic loops might consist of fluid-air levels (Patel and Hughes, 1992). Colonic haustrations show transverse thickening due to myxoid material containing submucosal infiltration (Desai et al., 2020). An ileus may arise because of coexistent infection (Thakur et al., 2020). It is difficult to determine the incidence of megacolon and ileus. The underlying mechanism behind megacolon pathogenesis is not very clear. Some of the cases suggest neuropathy, while infiltration of mucoid material and mast cells have been suggested in some cases causing separation of the muscularis propria fibers that results in muscle degeneration (Wells et al., 1977; Desai et al., 2020). Progressive muscular atrophy causes colonic atony in advanced stages that can be fatal (Yaylali et al., 2009). Colonic pressures are of reduced amplitude than in the control individuals (Kubiszewski et al., 2023). Patients showing increased pressures with bethanechol chloride and muscarinic drug respond clinically to thyroid hormones, suggesting that the power of contraction is retained by their muscles (Patel and Hughes, 1992). Individuals showing no bethanechol response are also not responsive to any other therapy. Blood flow may be compromised by bowel distention, causing ischemia (Riaz et al., 2020). Pseudo-obstruction is rarely observed and are usually linked to myxedema coma or severe hypothyroidism (Santos Argueta et al., 2022). Individuals may be responsive to a treatment regime of intravenous triiodothyronine (T3), that is commonly used for treating myxedema coma (Santos Argueta et al., 2022). Unrecognized hypothyroidism may result in increased colonic surgery morbidity or may lead to fatal outcome causing unnecessary procedures (Bergeron et al., 1997). Subclinical hypothyroidism may be turned florid due to surgical stress because of reduced peripheral thyroxine (T4) to T3 conversion (Hifumi et al., 2014). Pericardial and pleural effusions are frequently found associated with myxedema ascites, which are improved by thyroid replacement (Gomes Santos et al., 2023). High specific gravity and protein concentrations are characteristic of ascitic fluid (Gomes Santos et al., 2023). Ascitic fluid show relatively lower cholesterol concentration although myxedema is also characterized by hypercholesteremia. Myxedema may be further complicated by congestive cardiac failure; however, this can be factored out in many hypothyroidism patients with ascites. In these patients, the ascite pathogenesis is not clearly understood but can be linked to enhanced capillary permeability (Ipadeola et al., 2013).

Although the mechanism behind hypothyroidism-associated GI dysmotility is not clearly understood, glycosaminoglycans such as hyaluronic acid accumulation is caused by hypothyroidism in the smooth muscles and interstitial tissues of gastrointestinal tract resulting in delayed bowel transit (Maser et al., 2006). Reduced contraction amplitude and low LES pressure are characteristic of esophageal dysmotility, causing heartburn or dysphagia (Maser et al., 2006). Hypothyroidism is not characterized by any specific radiologic, endoscopic, or manometric, features (Chiovato et al., 2019). Besides thyroid function test, colonic transit study, abdominal X-ray, gastric scintigraphy, and hydrogen breath test may be performed to detect the condition while the clinical presentation would determine the specific treatment (Chiovato et al., 2019).

## Hyperthyroidism and GI motility

Sixty to eighty percent of thyrotoxicosis is associated with Grave’s disease (Grixti et al., 2024). It presents with tremor, hyperactivity, palpitations, tachycardia, heat intolerance, and weight loss. Nausea/vomiting, abdominal pain, and bowel habit change are some of the GI manifestations (Grixti et al., 2024). There may also be an association with other autoimmune disorders such as ulcerative colitis, and pernicious anemia (Zulfiqar and Andres, 2017; Zhao et al., 2024). Hyperthyroid symptoms may also correlate with dysphagia (Gunsar et al., 2003). In hyperthyroidism individuals, gastric emptying can be delayed, rapid or normal (Gunsar et al., 2003; Kisakol et al., 2003). No correlation is found in the tachygastria EGG findings or enhanced slow wave activity (Gunsar et al., 2003; Wang et al., 2019). In studies involving hyperthyroidism patients, no significant differences were observed between the patients and control group in terms of their solid phase gastric emptying (Kisakol et al., 2003; Daher et al., 2009). In another study, individuals on liquid meal showed accelerated gastric emptying (Kalra et al., 2014). Other studies observed delayed gastric emptying (Pfaffenbach et al., 1997; Gharahbaghian et al., 2007). Euthyroidism restoration results in slightly increased gastric emptying rate (Jonklaas, 2022). Hyperthyroidism treatment may improve abnormal myoelectrical activity and GI symptoms (Gunsar et al., 2003). Hyperthyroidism results in reduced acid secretion with histamine-resistant achlorhydria as shown by 16% of such patients (Dotevall et al., 1967; De Leo et al., 2016). Another study in contrast, showed no hyperthyroidism-associated acid secretion abnormality notwithstanding the fact that thyroid disease treatment caused increased acid secretion (De Leo et al., 2016). High levels of circulating thyroid hormones may be partly responsible for the reduced acid output, and it may necessarily not correlate with the duration or severity of thyroid disease (De Leo et al., 2016). Since a good number (33%–37%) of thyrotoxicosis patients show antiparietal cell antibodies, a likely autoimmune mechanism may also exist (Chiang et al., 2019; Boutzios et al., 2022). These antibodies in response to histamine could result in reduced maximal acid output (Boutzios et al., 2022). These antibodies, when present does not necessarily correlate with antithyroid antibody presence, the duration or severity of thyroid disease, or age (Boutzios et al., 2022). Histamine injection results in low pepsin secretion in such individuals, although the basal levels of pepsin remain normal (Ghosh et al., 2011). Eleven out of twenty-four hyperthyroid patients show hypergastrinemia, while 7 patients show autoimmune atrophic gastritis in one particular study (Wiersinga and Touber, 1980). The underlying mechanism of hypergastrinemia in such patients was attributed to β receptor hypersensitivity to catecholamines, and reduced acid production, or to some extent to enhanced levels of plasma catecholamine (Dacha et al., 2015). In contrast, one study showed no gastrin level changes (Miller et al., 1980). On the other hand, positive correlation was found between T3 and gastrin levels, and a negative correlation between T3 and acid output (Wiersinga and Touber, 1980). Treatment resulted in reduced levels of plasma gastrin in some patients, while it led to increased or reduced acid output in others.

In hyperthyroid patients, pancreatic and gastric responses are normal in coordination and quantity to a mixed nutrient meal (Miller et al., 1980). Delivery of nutrients to the duodenum from the stomach as well as the trypsin output following a meal was found normal when measurements were taken at the Treitz ligament. In contrast, another study found reduced output of trypsin in the chyme when measurements were taken 55-cm distal to Treitz ligament (Wiley et al., 1978). Hyperthyroid patients show changed composition and reduced bile output (Gunsar et al., 2003), however, the concentration of luminal bile acid is found higher than the critical concentration of micelle (Miller et al., 1980). Increased stool frequency is found associated with hyperthyroidism (Daher et al., 2009). In one study, diarrhoea was developed in 3 out of 7 patients, while 7 patients complained of increase in bowel movement numbers (Thomas et al., 1973). Some study, however, reported increased constipation incidences than diarrhoea (Maser et al., 2006). Hydrogen breath tests or small bowel barium studies reveal an enhanced mouth to cecum transit time which correlates negatively with T3 concentrations in the blood (Thomas et al., 1973; Tobin et al., 1989; Wegener et al., 1992; Daher et al., 2009). However, the gastric emptying contribution was not followed in those studies. Hyperthyroidism treatment with propylthiouracil normalized the transit time from mouth to cecum and resolved diarrhoea (Papa et al., 1997). Excretion of excess fecal fat is commonly observed in hyperthyroidism, which may be due to excessive fat ingestion through diet since the fat absorption coefficient is found normal in those individuals (Thomas et al., 1973). Dietary fat restriction led to reduced steatorrhea, while hyperthyroidism treatment lowered hyperphagia and excretion of excess fecal fat (Thomas et al., 1973; Papa et al., 1997). When on similar high-fat diets, hyperthyroid patients excreted significantly excess fecal fat as compared to normal individuals, ruling out hyperphagia as the only mechanism. The adrenergic system may partly be responsible for these abnormalities, as intake of propranolol reduced excretion of excess fecal fat and bowel movement numbers but increased small bowel barium transit time (Thomas et al., 1973). However, another study using lactulose breath test showed propranolol to have no effect on orocecal transit time, although in 1 patient diarrhoea was resolved (Daher et al., 2009). Hyperthyroid rats show development of reversible lactose intolerance, owing to deficiency of lactase (Thomas et al., 1973). However, small bowel biopsy, D-xylose absorption test, B12 and secretin test are found normal (Thomas et al., 1973). In one study, elevated levels of urinary D-xylose was observed in thyrotoxic patients following either intravenous or oral administration of the sugar because of an increase in renal excretion (Groener et al., 2013). No specific radiologic, endoscopic, or manometric GI dysmotility features are found in association with hyperthyroidism. Besides, thyroid function tests, breath and serum electrolyte tests have been found useful in measuring the orocecal transit time and in the diagnosis of periodic paralysis.

## Clinical manifestations associated with changes in GI motility

### Constipation

Individuals with GI motility problems commonly complain of constipation. This happens irrespective of any changes in stool frequency or colon transit time (Mearin et al., 2016). Often the complain of acute constipation by patients, may be a chronic problem, and the definition of constipation may be different between patients and physicians. Typically, patients will describe constipation as difficulty in defection, hard stools, or infrequent stool frequency. When compared with younger individuals, older adults reportedly use more laxatives although there is no actual difference in their stool frequency (Harari et al., 1996). Some patients suggest a need to strain rather than irregular stool frequency for their constipation. It remains to be found out whether the symptom-based subgroup identification can define pathophysiology in such groups. Medications may partially play a role in constipation development in some individuals (Gustafsson et al., 2019). Development of constipation is often associated with diet. In some individuals, constipation results in reduced meal frequency, fiber intake couldn’t be differentiated between non-constipated and constipated individuals (Yamada et al., 2021).

### Gastroesophageal reflux disease (GERD)

GERD presents itself as a chronic disorder and several studies suggest that individuals with severe GERD show erosive esophagitis and enhanced complication frequency (Cho et al., 2005). So, it is expected that increased disease duration results in higher complications. GERD pathogenesis depends greatly on lower esophageal sphincter competence, gastric emptying, and esophageal clearance. Several environmental and physiological factors result in greater complication incidences, that include gastric emptying, sedentary lifestyle, altered salivary bicarbonate secretion, and greater medication use leading to reflux disease, and reduced esophagus sensation to noxious stimuli. A changed esophageal motility, results in impeding the reflux acid clearance from distal esophagus (Lin et al., 2019). Greater GERD duration causes increased erosive esophagitis, strictures, ulceration, and Barrett’s esophagus (Lin et al., 2019; Adanir et al., 2021). Besides, the typical GERD symptoms, reflux disease needs consideration in patients with nausea/vomiting, chronic cough, wheezing, and anorexia.

### Bacterial overgrowth

Normal GI motility in healthy individuals restricts bacterial overgrowth (Dukowicz et al., 2007). Small intestinal bacterial overgrowth (SIBO) is referred to increased microorganism levels in the intestinal aspirate above 106 colony-forming units/mL. This refers to small intestinal colonic-type bacteria (Ponziani et al., 2016). Mechanisms regulating the growth of enteric bacteria when disturbed, causes SIBO. Small GI dysmotility has been found to be a very common underlying mechanism. Thus, change in small GI motility is a major risk factor for SIBO development (Ahmed et al., 2023). Impaired immunity, gastric acid barrier failure, anatomic alterations constitute some of the other risk factors (Adike and DiBaise, 2018). GI dysmotility presents the functional GI disorder, irritable bowel syndrome (IBS) (Peralta et al., 2009). Regardless of their underlying complaint, bloating is one of the symptoms in IBS patients. Bloating in IBS can be caused by SIBO. Peralta et al. (2009) through positive lactose breath tests observed SIBO in 56% of patients. Symptoms like abdominal discomfort, bloating, and diarrhoea in IBS can be caused by SIBO. More than 50% of hypothyroidism patients reportedly show with the presence of SIBO (Almandoz and Gharib, 2012). Lauritano et al. (2007) demonstrated that in hypothyroidism patients, a significantly higher (54%) number of subjects show presence of SIBO as observed through glucose breath test. Hypothyroid patients rarely report for diarrhoea but very commonly complain of constipation. In a study, a hypothyroidism-presenting young woman was predominantly reported for chronic diarrhoea (Goldin and Wengrower, 1990). Positive response to antibiotics and positive hydrogen breath test in such patients are strong predictors for SIBO. A successful antibiotic treatment and positive hydrogen breath test were observed in this patient (Goldin and Wengrower, 1990). In such patients, GI hypomotility-induced bacterial overgrowth can be the reason behind diarrhoea (Goldin and Wengrower, 1990). Thus, in hypothyroidism patients chronic GI symptoms can be a result of GI hypomotility-induced bacterial overgrowth. A common reason behind SIBO may be hypothyroidism (Lappinga et al., 2010). Hence, an evaluation for SIBO needs to be done in hypothyroidism patients complaining of chronic GI problems.

### Therapeutic approach

Symptoms of GI dysmotility may be resolved through the regulation of thyroid disease through surgery or medication (radio-iodine therapy, anti-thyroid drugs, e.g., carbimazole) as per disease indication (Nightingale et al., 2020). GI symptoms may be controlled using prokinetics, while beta-blockers may be useful in controlling diarrhoea (Nightingale et al., 2020). However, hormone replacement therapy is the most common therapeutic approach in patients presenting with symptoms of thyroid disorder-mediated GI dysmotility (Canpolat et al., 2013).

### Thyroid hormone replacement

A significant number of thyroid disorder patients with gastric dysmotility show improvement following thyroid hormone replacement (Canpolat et al., 2013). Data indicate that L-thyroxine therapy can improve the symptoms of gastric dysmotility that are apparent in the subclinical hypothyroidism (SCH) state. Hypothyroid patients are routinely treated worldwide with levothyroxine sodium monotherapy in replacement mode (Jonklaas, 2022). There has been strong suggestion for an individually tailored dose (Yin and Chen, 2013). Unfortunately, a high number of patients do not respond clinically or biochemically to thyroxine and only a large thyroxine dose is required to get the optimum concentration of serum TSH (Jonklaas, 2022). Also, suboptimal treatments for longer period adversely affect body homeostasis (Ettleson et al., 2022). Continued diagnostic procedures and frequent dose changes in such patients lead to increase in health costs (Ernst et al., 2017). There has been a review about the reasons behind the requirement of increased thyroxine (Virili et al., 2019). Among those, altered T4 absorption owing to modified gastric physiology comes up as one of the critical reasons (Centanni et al., 2006; Seng Yue et al., 2015). Although the underlying mechanism for impaired thyroxine absorption in gastric disorder patients is not very clear, but it seems related to both the physical and chemical properties of salificated as well as naïve thyroxine molecules (Dratman and Martin, 2020). Levo-isomer of thyroxine, levothyroxine is usually insoluble in most organic solvents as well as in water (Dratman and Martin, 2020). Sodium salt of thyroxine produced by using excess of sodium hydroxide is used in most pharmaceutical preparation as the final compound (Mateo and Hennessey, 2019). The preferred administration of the drug is oral owing to patient preference and safety (Mateo and Hennessey, 2019). Levothyroxine is not completely absorbed orally with only 70% of the dose administered is reported to be absorbed (Hays, 1991). In humans, the jejuno-ileal tract is the main site of oral thyroxine absorption while duodenum absorbs a few parts, but nothing has been reported to be absorbed in the large bowel unlike in the rats (Hays, 1988; Hays, 1991). Besides, absorption in the stomach has also been ruled out due to the lag time between appearance of thyroxine in the plasma and its ingestion (Hays, 1988). However, oral thyroxine bioavailability might be impacted by gastric physiology variations, leading to increased drug need. Nonetheless, thyroxine hormone replacement therapy can have beneficial effect in patients presenting with thyroid disease-related GI motility problems.

### Thyroid hormone absorption is improved by GI therapies

GI health improvement should be critically considered to improve absorption of thyroid medication before any attempts being made in fine-tuning thyroid disease type (Virili et al., 2019). For example, use of probiotics leads to reduced levels of TSH, lower the required dose of levothyroxine, and improve hypothyroid patients’ fatigue levels who continue to show symptoms even after optimum levothyroxine therapy (Talebi et al., 2020). In addition, there are studies that show improved levels of TSH upon *Helicobacter pylori* treatment that lower the required dose of levothyroxine (Centanni et al., 2006; Ribichini et al., 2017). A case study highlights the medication-related association between SIBO and thyroid showing the usefulness of targeting this linkage as therapeutic opportunity although no clinical trials have been carried out to this regard (Bohinc Henderson, 2021). The patient in question could not achieve optimal TSH balance even after multiple medication changes over a 6-month duration. Her symptoms could only be resolved and euthyroidism achieved when she was treated with rifaximin for SIBO besides receiving liquid T4 (Bohinc Henderson, 2021). Other work has documented similar successful results (Ruscio et al., 2022). Malabsorption of thyroid medication has also been improved with treatment for food sensitivities/intolerances (Asik et al., 2014). Seventy-six percent of hypothyroid patients show lactose intolerance, a lowered TSH was observed when such patients are provided a lactose-free diet (Asik et al., 2014). Similarly, individuals suffering from atypical celiac disease required lesser dose of levothyroxine when provided with a gluten-free diet (Virili et al., 2012). Optimal absorption of thyroid replacement requires a proper hydrochloric acid output. Several studies demonstrate the requirement of higher levothyroxine doses in patients with stomach acid secretion impairment (Centanni et al., 2006; Ribichini et al., 2017). Together, it can be inferred that GI health improvement (e.g., small bowel and gastric health) can lead to improved absorption of thyroid medication thereby lowering the overall medication need in many patients to maintain euthyroidism.

## Conclusion

**FIGURE 2:**
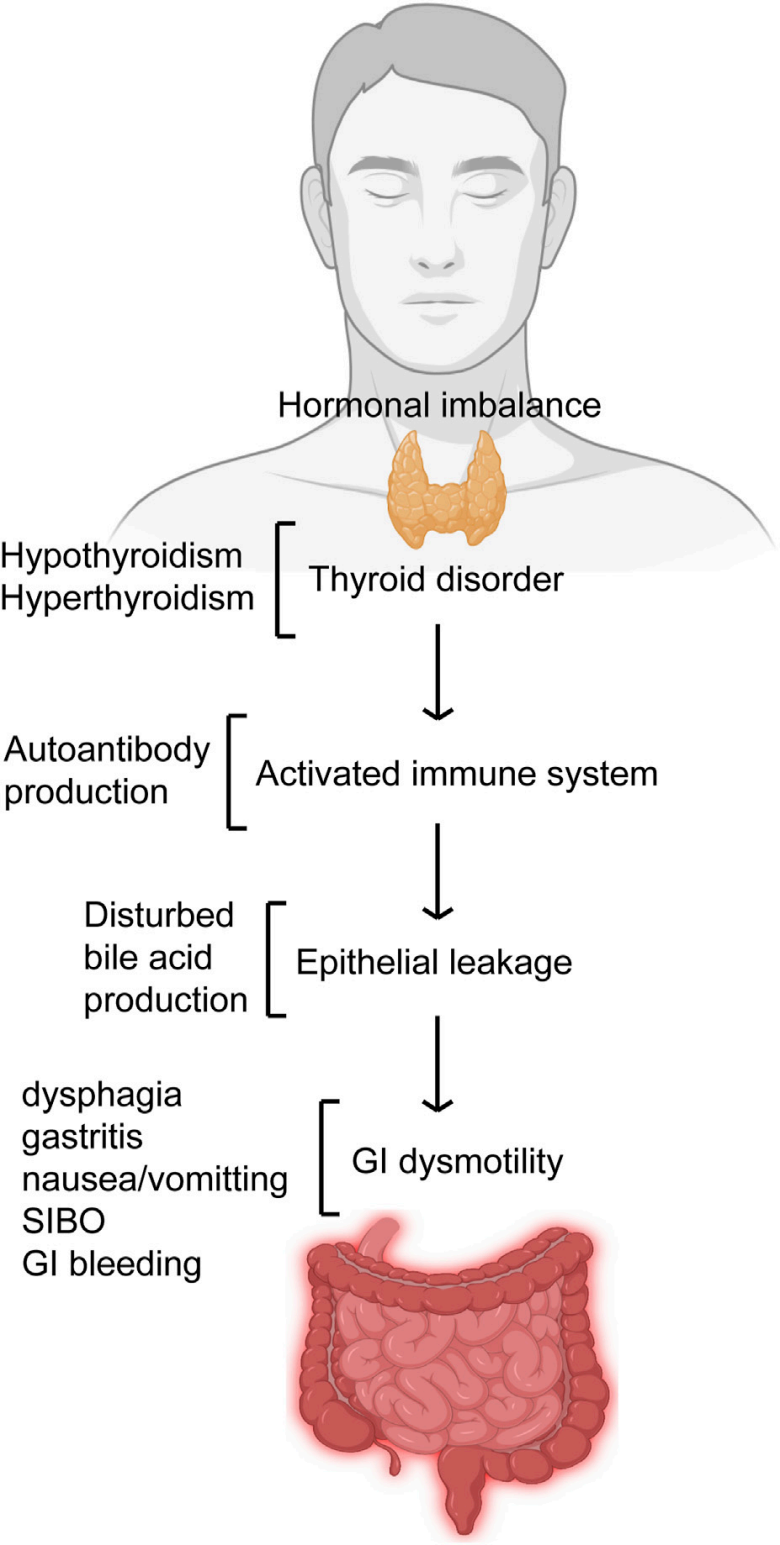
Thyroid disorder affects GI motility. Thyroid disorders like hypo- and hyperthyroidism that deregulates its hormonal balance results in imbalances like autoantibody production and bile acid production thereby leading to GI dysmotility. Impaired GI motility presents itself with features like gastritis, dysphagia, nausea/vomiting, GI bleeding and SIBO.

There has been an interest on the relationship between GI and endocrine functions. However, our knowledge about this relationship is very limited. As the endocrine function-mediated GI health gets gradually unfolded, there is dearth of scientific literature regarding the effect of endocrine function on GI motility. The impact of the thyroid on GI tract may vary depending on the involvement kind and the overall hormonal disturbance-associated effect (Figure 2). Nonetheless, some of the actions of thyroid on GI tract functions is widely accepted. There is no question regarding the influence of thyroid gland on the GI physiology. However, the relationship of thyroid disorder with a dysfunctional GI tract motility can be more intricate than it is thought. For example, there is lack of definite knowledge as whether the hormonal disturbances in thyroid disorders exert a direct effect on the GI dysmotility and related problems or these are autonomic nervous system-mediated secondary responses. Some suggestions can be found in the literature regarding the causal effect of the relationship between thyroid function and GI motility. Some of the suggested modes of actions include thyrotoxicosis-mediated direct effect on musculature, vagus effects, and hormonal-metabolic effects. A close association has been found between thyroid disorders and GI motility. Thyroid disorders prominently impair GI health including motility and as such evaluation of thyroid functions should be carried out in patients complaining of GI problems.
